# Rising Demand for Healthy Foods-Anthocyanin Biofortified Colored Wheat Is a New Research Trend

**DOI:** 10.3389/fnut.2022.878221

**Published:** 2022-05-09

**Authors:** Monika Garg, Satveer Kaur, Anjali Sharma, Anita Kumari, Vandita Tiwari, Saloni Sharma, Payal Kapoor, Bhawna Sheoran, Ajay Goyal, Meena Krishania

**Affiliations:** ^1^National Agri-Food Biotechnology Institute, Mohali, India; ^2^Panjab University, Chandigarh, India; ^3^Chitkara University School of Engineering & Technology, Chitkara University, Solan, India; ^4^Center of Innovative and Applied Bioprocessing (CIAB), Mohali, India

**Keywords:** black wheat, blue wheat, purple wheat, anthocyanins, health benefits, antioxidants, agronomy

## Abstract

Wheat is a vital and preferred energy source in many parts of the world. Its unique processing quality helps prepare many products such as bread, biscuit, pasta, and noodles. In the world of rapid economic growth, food security, in terms of nutritional profile, began to receive more significant interest. The development of biofortified colored wheat (black, purple, and blue) adds nutritional and functional health benefits to the energy-rich wheat. Colored wheat exists in three forms, purple, blue, and black, depending upon the types and position of the anthocyanins in wheat layers, regulated by the bHLH-MYC transcription factor. Colored wheat lines with high anthocyanin, iron, and zinc contents showed antioxidant and anti-inflammatory activity and possessed desirable product-making and commercial utilization features. The anthocyanin in colored wheat also has a broad spectrum of health implications, such as protection against metabolic syndromes like obesity, diabetes, hypertension, and dyslipidemia. The idea of developing anthocyanin-biofortified wheat shapes human beings' lifestyles as it is a staple food crop in many parts of the world. This review is a compilation of the currently available information on colored wheat in the critical aspects, including biochemistry, food processing, nutrition, genetics, breeding, and its effect on human health. Market generation and consumer awareness creation are vital challenges for its exploitation as a function food on a large scale.

## Introduction

The green revolution in Asia tripled wheat production and turned countries like India from wheat importers to a state of self-sufficiency, reducing mortality and malnutrition (1). Recently, it turned from boon to bane in India with four times the target wheat stocks and ever-increasing wheat production (2). It is time to shift from high-yielding wheat to quality wheat, and biofortified colored wheat gives a new twist.

People across the world are becoming increasingly concerned about health and nutrition. However, higher prices are usually taking such foods backstage. Colored wheat has gained much interest, as wheat is a cheap energy source and anthocyanin addition gives it functionality (3). Mainly, colored wheat exists in three predominant colors, i.e., black, blue, and purple, for the types and position of the anthocyanins in wheat layers. Purple wheat contains anthocyanins in the pericarp layer, blue wheat in the aleurone layer, and black wheat is a mixture of the anthocyanins in the pericarp and aleurone layers (4, 5). Anthocyanins have strong antioxidant potential, protecting the cell from free radical damage by neutralizing and scavenging them (6). Several research publications reported the health-promoting effects of anthocyanins, including anti-inflammatory, anticancer, antidiabetic, antiaging, neuroprotective, and prevention of cardiovascular diseases (5–8). Colored wheat can be extensively exploited as a unique ingredient for producing value-added food products due to its positive qualities. Based on the potential of colored grains, numerous functional foods, such as chapatti (9), beer (10), biscuits (11), muffins (12), soy sauce, pasta (13), have been reported from this wheat. Although the area under cultivation for colored wheat is still significantly less, scientists from several countries have started exploring it. The emergence of research publications from over 60 institutes in 16 countries shows rising interest in colored wheat, which parallels the increasing demand for functional foods and nutraceuticals. Several researchers have done extensive studies to understand the origin and genetics of colored wheat (14), anthocyanin biosynthetic pathway genes, and transporters (15) and their regulation (16). Colored wheat has garnered interest as an alternative source of functional foods due to its high nutritional profile and ease of growing. This review compiles all available information on colored wheat in key aspects, including origin, genetics, biochemical composition, breeding, food processing, and health benefits.

## Origin and Genetics of Anthocyanin Accumulation in Colored Wheat

Commonly, wheat seeds do not exist in colored forms. Colored wheat originated either from different landraces or through a wide hybridization process. Colored wheat is known to exist in three different forms: purple, blue, and black, depending upon the types and position of the anthocyanins in wheat layers (4, 17).

**Figure 1:**
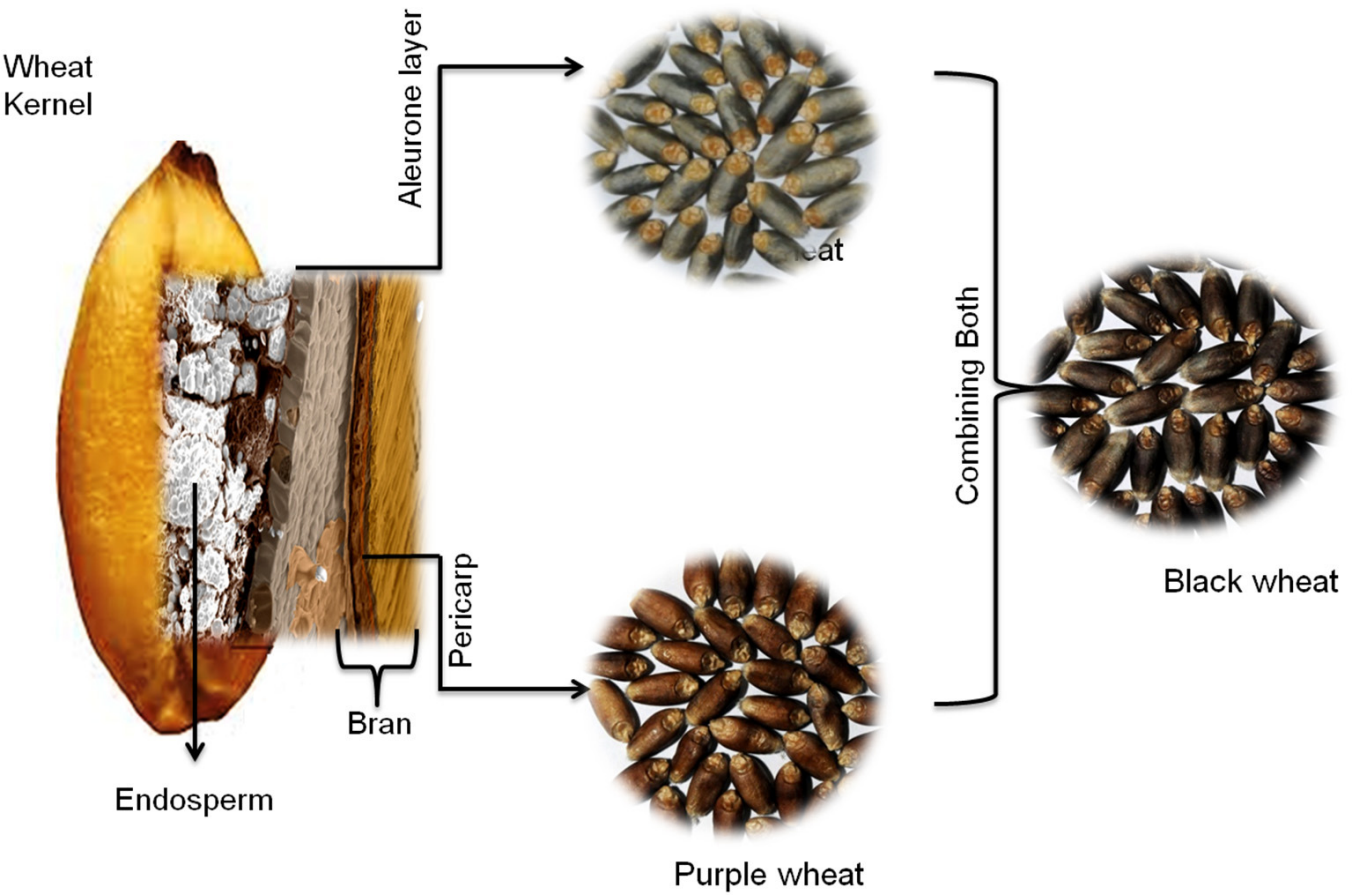
Localization of anthocyanins in different layers of wheat seed. Purple wheat contains anthocyanins in the pericarp layer, blue wheat has anthocyanins in the aleurone layer, and black wheat is a mixture of both, with anthocyanins in both the pericarp and aleurone layers.

In the purple wheat, pigmentation is present in the pericarp layer (4, 17–19) (Figure 1). Its origin traces back to Ethiopian purple tetraploid Abyssinian wheat (*Triticum aethiopicum*) (20). It was first collected by Wittmack in Abyssinia (Northern Ethiopia) in the early 1870s and then introduced to Europe, where botanists widely distributed it. Later, commercial cultivars of purple wheat were released in countries like Australia, China, New Zealand, Canada, India, and several European countries. Landraces of purple wheat are still cultivated in Tigray, Ethiopia.

The genetics of the purple pericarp is complicated as three dominant alleles control it; *Pp-1* located at chromosome 7BL designated as *Pp-B1* (7B of *T. durum*, 7S of *Ae. speltoides*), *Pp-D1* located at chromosome 7D (*T*. *aestivum*), and *Pp3* located at chromosome 2A (21). Thus, two complementing components are necessary to develop the purple pericarp characteristic, situated in distinct genomes, namely A and B or A and D. Even if two other genes (Pp-B1 and Pp-D1) are dominant, the grain will not be purple in the presence of the recessive *Pp3* gene. Therefore, the purple pericarp trait is expressed only in the allopolyploid wheat and not diploid progenitors. Recently, *Pp-A1* located on 7AL imparted light purple color in the presence of the dominant *Pp3* gene (2A) (14). Shoeva et al. (22) identified that *the Pp3* (2A) gene coded for bHLH-MYC regulatory transcription factor. Jiang et al. (16) proposed that two transcription factors, i.e., R2R3-MYB and bHLH-MYC, co-regulate the anthocyanin synthesis in the purple pericarp trait, but the actual mechanism controlling this trait is still obscure.

The blue color of wheat is because of pigmentation in the aleurone layer (Figure 1) (4, 17–19). The introgression process introduced this trait into wheat (*T. aestivum*) from various wild relatives of wheat (20). It was first time reported as a European Blaukorn strain used as marker lines in breeding work. The blue aleurone trait in this material originates from the einkorn wheat (23). Subsequently, several blue lines were developed/selected that had introgression of chromosomes (substitution line) or part of a chromosome (translocation lines) from wild wheat *Agropyron tricholphorum, Ag. Glaucum*, or *Ag. elongatum* in North America (23) and *Triticum boeoticum* in Poland (24). The blue aleurone trait is known to be controlled by genes originating from different wild species; *Ba1* {syn. *Ba*(*b*)}, a dominant gene located at chromosome 4AgL of *Ag. elongatum, Ba2* {syn. *Ba*(*a*)}, an incompletely dominant gene located at the 4A chromosome of *T. monococcum*/*T. boeoticum, BaThb* {syn. *Ba*(*c*)}, a dominant gene located at 4J of *Th. Bessarabicum*, and *BaThe* {syn. *Ba*(*d*)} from the 4E chromosome of *Th. elongatum/ Th. ponticum* (25, 26). Li et al. (27) proposed that the bHLH transcription factor (*ThMYC4E*) controls this trait in *Th. ponticum* 4E chromosome addition line. However, the actual molecular mechanism behind the blue color trait is not yet clear.

Chinese researchers initially developed black wheat by hybridizing purple pericarp and blue aleurone wheat (28, 29). It was also developed in Japan and later transferred to the Indian germplasm (4). Black wheat development has been reported in Austria (30) and Ukraine (31). It appeared black (deep purple hue) as it had color in both pericarps and aleurone layers (Figure 1) and has the same genetics as purple and blue wheat.

Anthocyanins are formed in the endoplasmic reticulum and stored in the vacuoles (15). The regulation of anthocyanin biosynthesis has been an area of significant interest. Genes involved in the anthocyanin biosynthesis pathway are regulated by ternary MYB-bHLH-WD40 complexes (MBW) formed by R2R3-MYB transcription factors, bHLH transcription factors, and WD40 proteins (32, 33). Jiang et al. (16) revealed their cooperation to modulate anthocyanin production in the wheat purple pericarp. They reported that TFs, MYB 1, and bHLH co-regulate anthocyanin biosynthesis in purple pericarps of wheat. MYB-bHLH-WD40 (MBW) ternary complexes that regulate anthocyanin production in monocots have not been reported in wheat and still need exploration.

## Biochemical Composition of Colored Wheat

Colored wheat grains are a rich source of macronutrients such as carbohydrates, fats, and proteins and micronutrients like anthocyanins, vitamins, minerals, and carotenoids (17). Several reports have also characterized these compounds in colored wheat to assess their functionality.

### Macronutrients

Among the macronutrients, carbohydrate, starch is the main constituent distributed in the endosperm part of the seed. Black, blue, and purple wheat types have been shown to have comparable (34) or lower carbohydrate content than white wheat (9). Abdel-Aal et al. (35) reported lower starch content in purple wheat (54.4 %), similarly, Gamel et al. (36) and Kassegn (37) also reported lower starch content in purple wheat as compared to white wheat. In contrast, Kumari et al. (9) compared the carbs content of all three colored wheat (64–66%) to white wheat (68%). Various researchers have also explored the protein content. Regular wheat has 8–14% of protein content, while colored wheat has a similar () or higher protein content than white wheat (9, 38). A few observations on protein content include reports of 11.74– 18.17% higher protein content in blue and black wheat (39), 10.87, 12.08, and 12.25% higher in purple, blue, and black (9), 15% higher in purple (40), and 17% higher in black (41). Also, the colored wheat varieties possessed a better amino acid profile in the total amino acid content and nutrition index/essential amino acid index (39, 42). Colored grain varieties exhibited 8–18% higher amino acids than common wheat. Further, the essential amino acid contents were similar (42) or higher [(39) 7–18%]. Researchers also noted that the content of lysine (first restrictive amino acid) in colored wheat was either similar (42) or higher than that of common wheat (39). Moreover, lower amino acid cooking losses were observed compared to white wheat (42). Besides carbohydrates and proteins, fats and oils are equally essential nutrients in low amounts in wheat. The proximate composition of colored wheat indicated 0.3–2.6% fat content (9, 22, 43, 44) that was like the white wheat varieties. The starch and protein content varies with the genotype, location, soil fertility, and grain size.

### Micronutrients

Minerals like iron (Fe), calcium (Ca), and zinc (Zn), are vital health components, for example, Fe forms an integral part of hemoglobin, whereas Ca is essential for bones, and Zn helps in maintaining the overall mental health. The relative increase varies for mineral as well as color, e.g., Guo et al. (40) reported a 100% increase in Zn, Fe, Mg, and K in purple wheat, and Tian et al. (39) noted 108.54–142.68, 8.57–42.86, and 5.31–40.63% increase in Zn, Fe, and Mg, respectively, in different colored wheat. Colored wheat exhibited a comparatively rich micronutrient profile than white. It accumulates higher Fe and Zn in grains (34, 38–40, 45). Other essential minerals include magnesium (Mg) (39, 40) potassium (K) (40), and selenium (Se) (46, 47). In addition, colored wheatgrass (seedlings) also reported higher content of selected minerals, including Fe, Zn, Cu, Mg, and Mn (48).

Vitamins are another important micronutrient required by the body. Generally, wheat is considered a good source of the B group vitamins and vitamin E (tocols). The common white wheat varieties are reported to contain 20–40 μg g^−1^ total tocols (49), 2.6–6.1 μg g^−1^ of thiamine (vitamin B_1_), 0.5–1.1 μg g^−1^ of riboflavin (vitamin B_2_), and 1.5–3.2 μg g^−1^ of pyridoxine (vitamin B_6_) (50). Researchers compare colored wheat to white wheat, and the average vitamin content is much better in colored wheat. Purple and blue wheat lines show higher diversity of different tocols and vitamin E activity (50). Blue wheat has 5–36% higher vitamin E content (49), while purple has 0.32–57.83% (40). Higher vitamin B1, B2, and B9 were reported in purple and blue wheat lines (40, 50).

### Anthocyanins

**Figure 2:**
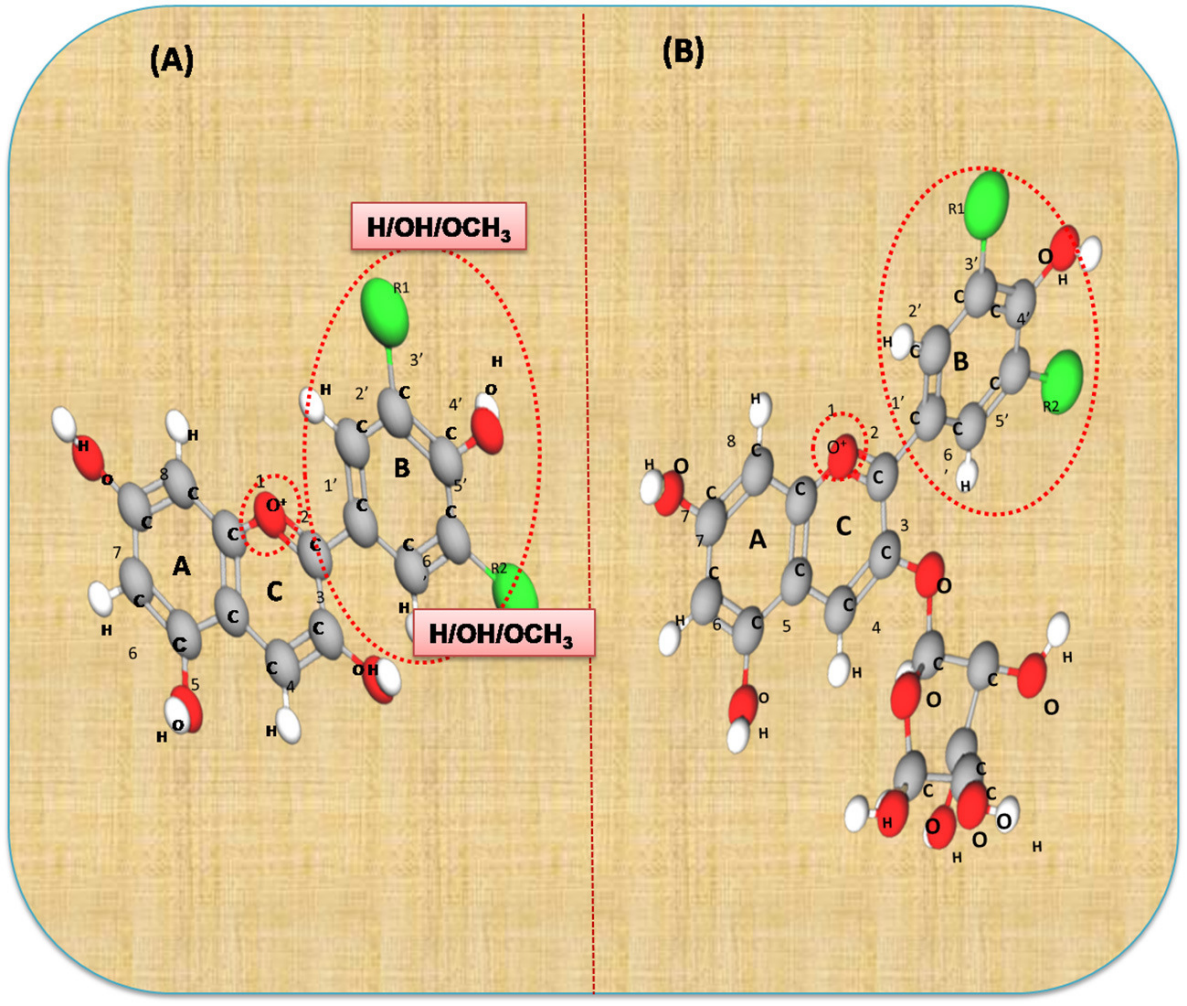
The basic structure of anthocyanidin **(A)** and anthocyanin-3-glucoside **(B)**. Anthocyanins comprise hydroxyl or methoxyl group on the B-ring of flavylium ion. The B-ring and positive charge at the oxygen atom of the C-ring (red dotted circles) is reactive which makes the anthocyanin molecule a strong antioxidant.

Anthocyanins are secondary plant metabolites recognized as natural pigments that provide red, violet, and blue color to fruits, vegetables, and cereal grains. Pelargonidin, cyanidin, delphinidin, peonidin, petunidin, and malvidin are well-known glycosides of anthocyanidins. Delphinidin gives the bluish color, whereas cyanidin and pelargonidin are accountable for the purple and red color in plants. Structurally, anthocyanins comprise hydroxyl or methoxyl groups on the B-ring of 2-phenyl benzo pyrylium or flavylium ion (Figure 2). This B-ring and a positive charge at the oxygen atom of the C-ring (oxonium ion) are reactive, making the anthocyanin molecule a potent antioxidant (51). The anthocyanidins are stabilized through hydroxylation, methylation, glycosylation, and acylation (51).

**Figure 3:**
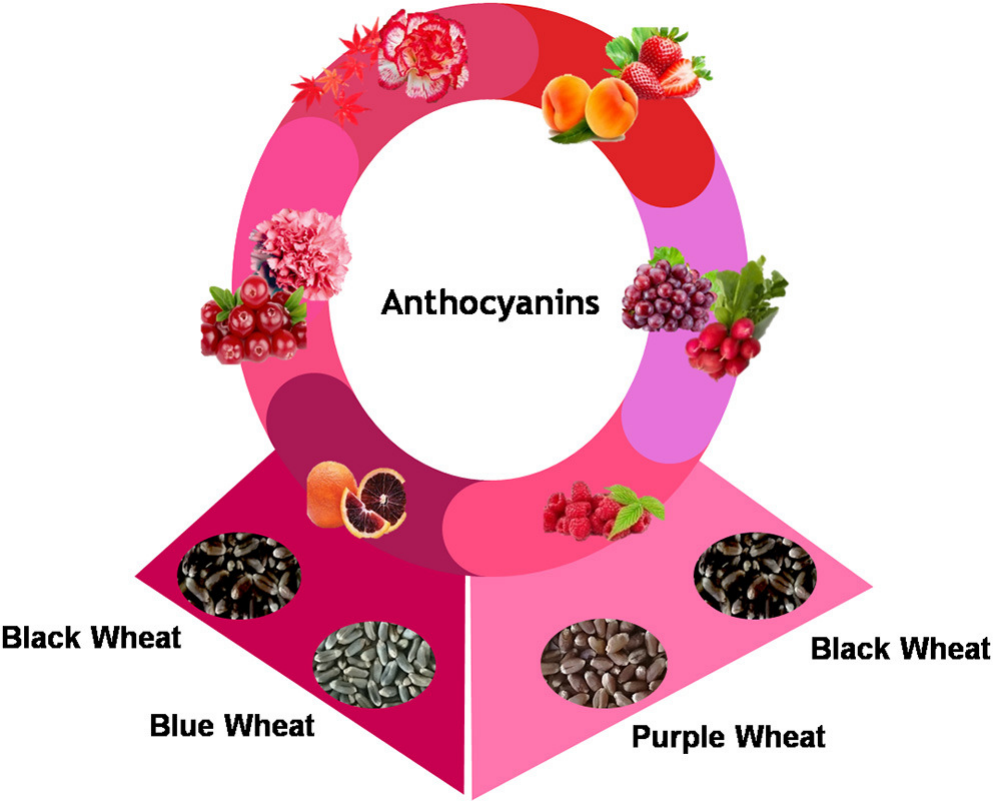
Six types of anthocyanins are commonly found in colored wheat. Purple wheat has a higher content of cyanidin derivatives, while blue has delphinidin, and black has both of them.

Many reports acknowledged that different colored wheat varieties have varying total anthocyanin content (TAC) levels, as listed in Table 1. TAC varies from 277 to 95, 278 to 22, 211 to 72, 10 to 7 mg/kg, and in black, purple, blue, and red wheat, respectively (9, 56, 57). Details of different anthocyanins reported in colored wheat are presented in Table 1. Out of the 11 forms of cyanidins shown in Table 1, four are acylated and present in black, purple, and blue wheat. Similarly, two acylated forms of delphinidin are present in the three colored wheat varieties. One form of acylated malvidin, pelargonidin, and peonidin was observed in the following wheat varieties: black, purple, and blue wheat; purple, blue, and purple wheat; and purple wheat. Cyanidin-3-glucoside is the dominant anthocyanin in purple wheat (17). Contrary to this, in blue wheat, delphinidin-3-glucoside (58) and delphinidin-3-galactoside (52) have been individually reported as the dominant anthocyanin by different research groups. Black wheat, a combination of purple and blue colors, has a high concentration of delphinidin and cyanidin derivatives, i.e., delphinidin-3-galactoside and cyanidin-3-glucoside (52) (Figure 3). These variations are also thought to be attributed to the genotypic and environmental conditions or could be because of the variation in extraction and quantification methods.

**Table 1:** Anthocyanin composition in colored wheat grains.

**Anthocyanidin name**	**Anthocyanins**	**Chemical formula and m/z**	**Wheat type**	**Amount (mg/kg)**	**References**
**Cyanidin** 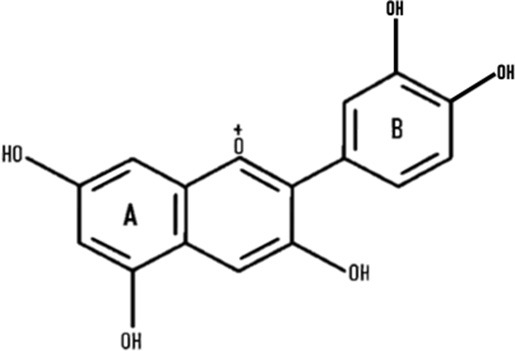	Cyanidin 3-glucoside	C_21_H_20_O_11_ 449,287	Purple	2.64, 10.3, 12.02, 52.80,103	(4, 17–19, 35, 52, 53)
			Black	20.50	
			Blue	3.07, 10.54, 10.76, 4.50	
			Red	4.02	
	Cyanidin-3-rutinoside	C_27_H_30_O_15_ 595,287	Purple	5.30, 32.53, 0.60	(4, 21, 52–54)
			Blue	8.42, 12.78, 13.84,0.20	
			Black	11.14	
	Cyanidin-3-arabinoside	C_20_H_19_O_10_ 419,287	Purple	25.1	(19)
	Cyanidin-3-rutinoside- 3'-glucoside	C_33_H_40_O_10_ 757,597,449,287	Blue	Dnq	(4)
			Purple	Dnq	
	Cyanidin-3-(6”- malonylglucoside)	C_24_H_22_O_14_ 535,287	Purple	31.08	(4, 18, 55)
			Black	Dnq	
	Cyanidin-3-galactoside	C_12_H_21_O_11_ 449,287	Purple	0.98, 72.0	(19, 55)
	Cyanidin-3-(3”,6”- Dimalonylglucoside)	C_27_H_24_O_17_ 621,287	Purple	8.15	(4, 55)
			Black	Dnq	
	Cyanidin-3- succinyl-glucoside	C_25_H_24_O_14_ 549,287	Purple	1.2	(4, 18, 21)
			Blue	Dnq	
			Black	Dnq	
	Cyanidin-3-(2-G-xylosylrutinoside)	727,287 C_32_H_38_O_19_	Blue	Dnq	(4)
			Black	Dnq	
	Cyanidin-3-(6”- feruloylglucoside)- 5-glucoside	C_37_H_38_O_19_ 787,287	Blue	Dnq	(4)
			Purple		
			Black		
	Cyanidin-3,5- di-glucoside	C_27_H_31_0_16_ 611,287	Purple	Dnq	(35)
Delphinidin 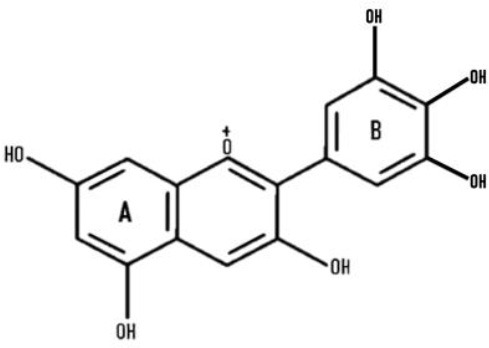	Delphinidin-3-arabinoside	C_20_H_19_ClO_11_ 435,303	Purple	16.7	(19)
	Delphinidin-3-galactoside	C_21_H_21_ClO_12_ 405,303	Purple	38.3, 0.40	(19, 52)
			Blue	4.95	
			Black	29.14	
	Delphinidin-3-glucoside	C_21_H_20_O_12_ 465,303	Blue	22.23, 29.03, 0.40	(4, 21, 52)
			Purple	0.08	
			Black	25.64	
	Delphinidin-3-rutinoside	C_27_H_30_O_16_ 611, 435, 303	Blue	29.65, 43.87, 33.44, 2.08	(4, 17, 19, 52)
			Purple	Dnq	
			Black	0.66	
	Delphinidin-3-(6” - malonylglucoside)	C_24_H_22_O_15_ 511,303	Purple	Dnq	(4)
			Black	Dnq	
	Delphinidin-3- caffeoylglucoside	C_30_H_26_O_15_ 627,465,303	Blue	Dnq	(4);
			Purple		
			Black		
**Malvidin** 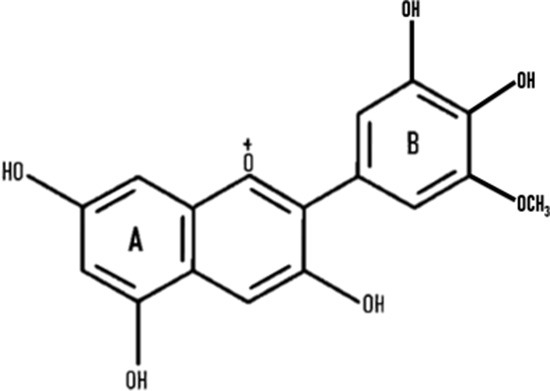	Malvidin-3-glucoside	C_23_H_24_O_12_ 493,331	Blue	12.04, 5.5	(4, 17, 19, 52, 53)
			Purple	0.48, 51.6, 1.32	
			Black	2.18	
			Red	0.22	
	Malvidin-3-rutinoside	C_29_H_34_O_16_ 639,493,331	Blue	2.0	(4, 18)
			Purple	Dnq	
			Black	Dnq	
	Malvidin-3-(6”-p-caffeoylglucoside)	C_32_H_30_O_15_ 655,493,331	Blue	Dnq	(4)
			Purple	Dnq	
			Black	Dnq	
	Malvidin-3-rutinoside-5-glucoside	C_35_H_44_O_21_ 801,639,493,331	Blue	Dnq	(4)
			Purple	Dnq	
			Black	Dnq	
Pelargonidin 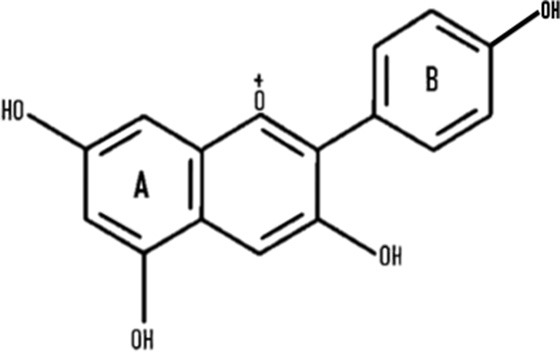	Pelargonidin-3-galactoside	C_21_H_21_ClO_10_ 433,271	Purple	26.1	(19, 53)
	Pelargonidin-3-arabinoside	C_20_H_19_O_9_ 403,271	Purple	9.3	(19, 53)
	Pelargonidin-3-glucoside	C_21_H_20_O_10_ 433,271	Blue	0.39	(4, 19, 52, 53, 55)
			Purple	28.8, 2.58, 1.88	
			Black	2.13	
	Pelargonidin-3-(6”- malonylglucoside)	C_24_H_22_O_13_ 519,271	Blue	trace	(4, 55)
			Purple	Dnq	
	Pelargonidin-3-rutinoside	C_27_H_30_O_14_ 579,433,271	Blue	Dnq	(4)
			Purple	Dnq	
			Black	Dnq	
Peonidin 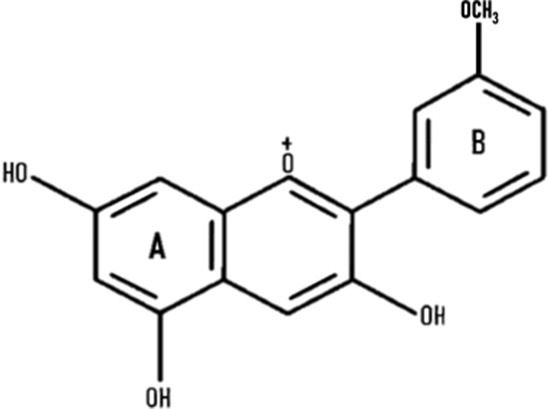	Peonidin-3-arabinoside	C_21_H_21_ClO_10_ 449,301	Blue	2.22	(17, 19, 21)
			Purple	9.3	
	Peonidin-3-glucoside	C_22_H_22_O_11_ 463,301	Blue	0.88	(4, 17–19, 52)
			Purple	4.61	
			Black	1.40	
	Peonidin-3-galactoside	C_22_H_23_ClO_11_ 463,301	Blue	1.94	(4, 17)
			Purple	0.58	
			Red	0.33	
	Peonidin-3-rutinoside	C_28_H_32_O_15_ 609,463,301	Blue	1.2, 0.76	(4, 18, 54)
			Purple	9.36	
			Black	0.97	
	Peonidin-3,5-diglucoside	C_28_H_32_O_16_ 625,463,301	Blue	0.31	(4, 52)
			Black	0.23	
			Purple	Dnq	
	Peonidin-malonyl-glucoside	C_25_H_25_O_14_ 549,301	Purple	0.6	(18, 35)
Petunidin 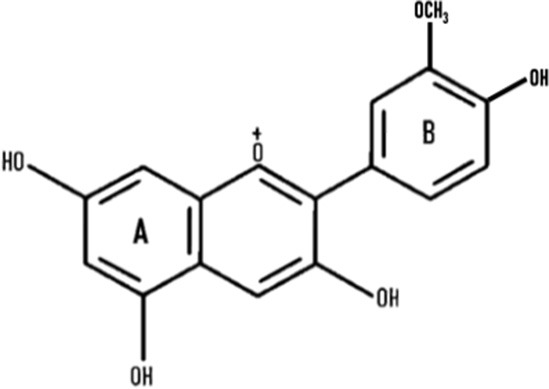	Petunidin-3-glucoside	C_22_H_22_O_12_ 479,317	Blue	3.18	(4, 18, 52)
			Purple	40.4	
			Black	2.29	
	Petunidin-3-rutinoside	C_28_H_33_O_16_ 625,317	Blue	4.5	(18)
	Petunidin-3-rutinoside-5-glucoside	C_34_H_42_O_21_ 787,625,479,317	Blue	Dnq	(4)
			Purple	Dnq	(4)
			Black	Dnq	
					

### Phenolics and Related Compounds

Phenolic compounds are secondary metabolites of plants, mainly recognized for their antioxidant activity. Phenolic acids include p-hydroxybenzoic, protocatechuic, vanillic, syringic, and gallic acids, as well as hydroxycinnamic acid (HCA) derivatives such as p-coumaric, caffeic, ferulic, and sinapic acids (29). Phenolic compounds are present in wheat as soluble-free and insoluble-bound forms, abundant in bran. Various studies reported up to 30% higher total phenolics content in black, purple, and blue wheat (8, 9, 57). Colored wheat lines reported higher content of different phenylpropane and flavonoid metabolites, including anthocyanins, flavones, flavonols, and flavonoids (57).

Soluble and insoluble phenolic acid profiles were compared between white wheat and colored wheat varieties and their product form, like in chapatti (Indian flattened bread). They found that pigmented wheat had more soluble and insoluble phenolic acids than white wheat (9). Colored wheat varieties (black, blue, and purple) have nine phenolic acids, i.e., gallic acid, p-hydroxybenzoic, caffeic, syringic, p-coumaric, vanillic, gentisic, o-coumaric acid, and ferulic acids (29). Ferulic, sinapic acid, p-coumaric, and vanillic acids are the most common phenolic acids reported in pigmented wheat cultivars. Like previous white wheat reports, ferulic acid was the most abundantly occurring phenolic acid in colored wheat (56, 59). Hence, colored wheat can be a promising source of TPC in addition to anthocyanins.

Micronutrient melatonin has a positive effect on brain health and acts as an antioxidant. Purple wheat has higher melatonin content than normal wheat (19). The carotenoids or yellow pigment content (YPC) has also been studied from colored wheat as these also act as antioxidants. Yellow durum wheat (tetraploid) with the highest YPC is used for pasta making. Lutein is the major carotenoid in colored and white wheat (60). YPC variation of hexaploid purple and blue wheat is from 3.3–7.6 ppm compared to 2.6 ppm for red wheat and 7 ppm from yellow durum wheat (61). In the case of purple durum wheat (7.4 ppm), the YPC content is as high as yellow durum wheat (7.7 ppm) (13). The total flavonoid content (TFC) is also considered an essential bioactive micronutrient due to its good antioxidant capacity and health benefits (62). The TFC in various varieties of wheat varies from 2 to 7 ppm. Colored germplasm was said to have more TFC in it. Liu et al. (56) reported that purple wheat contains the most elevated TFC at 2–10 ppm, whereas yellow, red, and normal wheat recorded 1.3, 1.1, and 0.9 ppm, respectively. Sun et al. (63) reported higher TFC in black wheat, followed by red and white wheat. Li et al. (8) compared TFC of black and purple wheat and found that it was high in black wheat compared to purple wheat. Wang et al. (57) reported a high level of protocatechuic and gentisic acids in purple and black wheat, whereas 4-methoxycinnamic and 3,4,5-trimethoxycinnamic acids were found to be much higher in yellow and blue wheat.

## Processing Quality and Commercial Utilization of Colored Wheat

Wheat has a unique processing property that allows it to be cooked into various food items such as bread, biscuits, chapattis, and noodles. Protein and starch are the primary components of wheat endosperm and play essential roles in determining its processing quality. Glutenin and gliadin, which give the dough its distinctive extensibility and elasticity, are found in wheat gluten protein, while amylose and amylopectin are in starch. Various environmental factors like temperature, irrigation, soil, day length, etc. along with the genotype, affect the grain constituents. The gluten index value of black-grained wheat is 69.74% which lies in the optimum range (60–90%) for making good-quality bread (41). It also has a low stickiness value and has the HMW-GS pattern of 2^*^ and 5 + 10, which means it has better baking properties. Kumari et al. (9) observed the dough extensibility and chapatti separation distance in different colored wheat varieties. Both colored and white wheat dough had medium extensibility, indicating their suitability for chapatti making. The extension distance of black and blue chapattis was higher than the others, indicating that black and blue wheat make soft and extensible chapatti. Sodium dodecyl sulfate sedimentation (SDSS) as an indirect measure of flour swelling power was in the order of white>purple>blue>black wheat. Lower SDSS is preferred for good-quality chapatti (9). Thus, colored wheat lines can be bred and utilized for specific product requirements. Colored wheat exhibits high nutritional components and high tensile strength, making it a prime ingredient for the bakery industry (34, 39).

Thus, colored wheat lines can be bred and utilized for specific product requirements.

**Figure 4:**
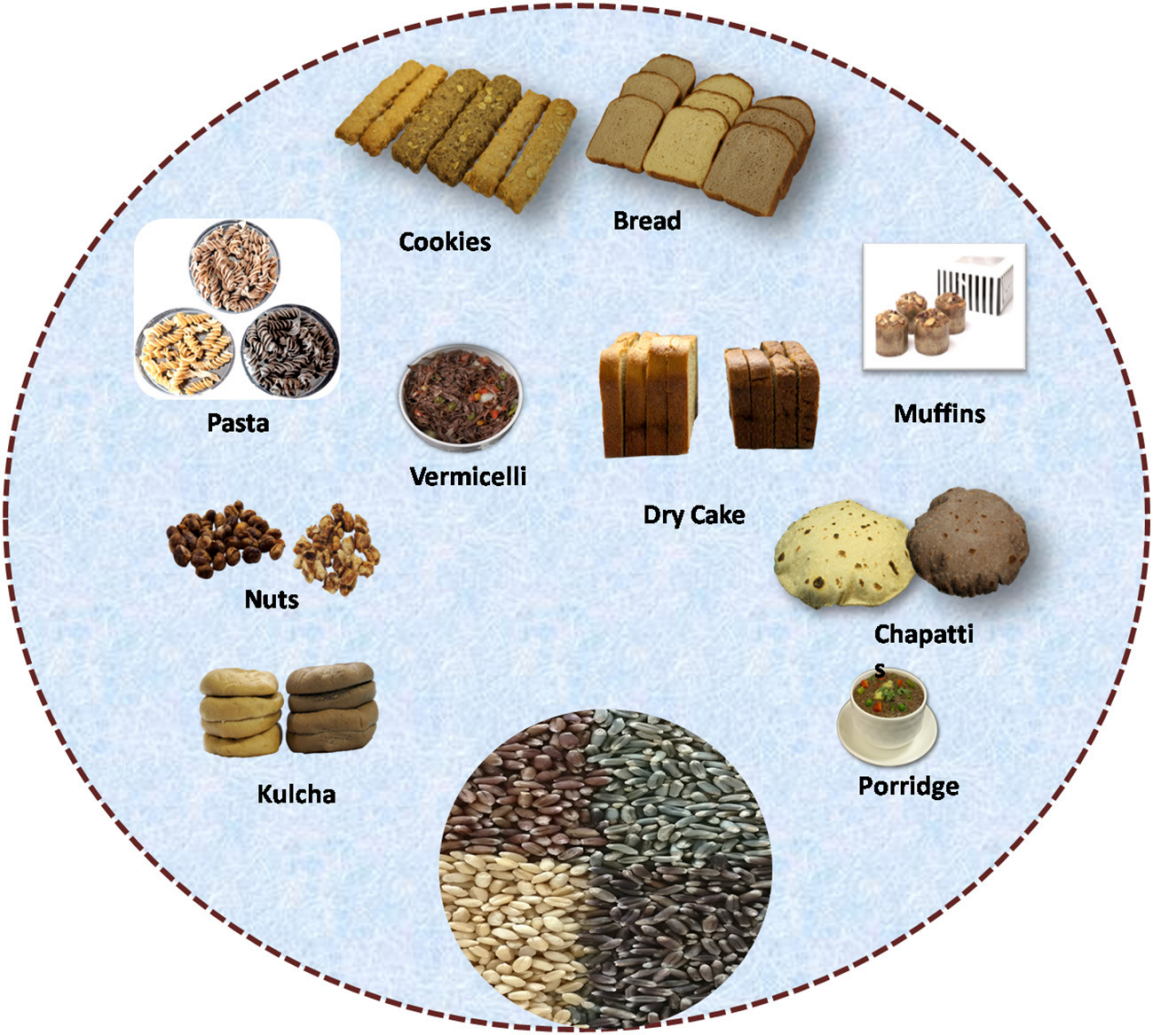
Development of different colored wheat products with high nutritional values.

Bread and bakery products from whole wheat have recently gained importance, being a rich source of dietary fiber and nutrients. However, bakery products prepared from white wheat flour have a low antioxidant capacity and are therefore fortified with colored wheat flour for improved nutritional benefits (Figure 4). The products prepared from colored wheat have high carotenoids, total dietary fiber (TDF), TPC, TAC, and high antioxidant activity (11, 13). Sharma et al. (34) reported that colored wheat lines have higher nutritional content and similar processing parameters than common wheat and, therefore, can be commercialized to make different functional foods (34).

In recent years, many food-processing researchers worldwide have come forward to exploit colored wheat for utilization in the food industry. Many researchers have reported about colored wheat high-value products like bread, biscuits, pasta, noodles, bars, and crackers, indicating their potential to substitute common wheat (64) (Figure 4). The examples include purple wheat muffins (12), antho-beer (10), blue wheat noodles, chapatti from black, blue, and purple wheat (9), biscuits (11), bread (65), and various other products such as vinegar, breakfast cereal, and instant noodles produced from black-grained wheat (**Table 4**). Interestingly, biscuits are among the most consumed bakery items having a longer shelf life; hence, colored wheat biscuits are a portion of healthy food. Pasqualone et al. (11) successfully demonstrated the use of purple wheat for producing anthocyanin-rich biscuits with higher antioxidant activity. Besides this, the usage of colored wheat in making bread with high antioxidant activity was also reported (65). Additionally, anthocyanin-rich fractions may be obtained from appropriate procedures prior to milling (66). These fractions are also rich in fiber and minerals and could represent suitable ingredients to produce staple foods such as pasta or bread. The presence of anthocyanin increased the shelf life of bakery products and their resistance to the development of mold under moist conditions (67). Therefore, colored wheat varieties contained all the features required for commercial product development, paving the way for their industrial utilization.

## Effect of Cooking on the Stability of Phytochemicals in Colored Wheat

Anthocyanins and phenolic acids stability is the primary aim of many current findings due to their great potential applications in the food and health sector (5–8). Modern food-processing technologies require high temperatures (160–300°C), and studies showed that anthocyanin stability is decreased in foods after thermal processing (12). Because of the health benefits, there is a need to preserve the content of anthocyanins and total phenolics during thermal processing. However, these are stable at lower temperatures, and their stability decreases with an increase in temperature and the heating duration (54, 68). Therefore, appropriate knowledge of unit operations is needed to convert raw materials into finished products.

Various parameters such as species, storage conditions, oxygen, milling, fermentation, roasting, blanching, cooking time, enzymatic reaction, cooking temperature, acidity levels, light, steaming, extrusion, pH, food ingredients, and interactions between macronutrients and micronutrients in finished products impact the stability of these bioactive compounds (64). Table 2 compiles cooking-induced changes in the anthocyanins, phenolics, carotenoids, and antioxidant activities of colored wheat lines.

**Table 2:** Cooking induced changes in the anthocyanins, phenolics, carotenoids, and antioxidant activities of colored wheat.

**Line**	**Product**	**Cooking method**	**Cooking time/temperature**	**Anthocyanins (%) (+ gain/– loss; %)**	**Phenolics (%) (+ gain/– loss; %)**	**Carotenoids (%) (+ gain/– loss; %)**	**Total antioxidant activity (+ gain/– loss; %)**	**References**
PW	Bread	Baking	25 min at 200 °C	−54.40	+35.1		+32	Yu and Beta (65)
BW	Bread	Baking	31 min at 180 °C	−42.6				Bartl et al. (54)
BW	Bread	Baking	21 min at 240 °C	−10				Bartl et al. (54)
PW	Bread	Baking	31 min at 180 °C	−72.6				Bartl et al. (54)
PW	Bread	Baking	21 min at 240 °C	−61				Bartl et al. (54)
PW	Bread	Baking		−62				Gamel et al. (43)
PW BW	Bread Bread	Dough making Dough making		−31 −10				Eliášová et al. (69)
PW BW	Bread Bread	Baking Baking	14 min at 240 °C	−2.7 −53				Eliášová et al. (69)
PW	Pancake	Baking	2 min at 250°C	−44.18				Gamel et al. (43)
PW	Porridge	Boiling	12 min at 100 °C	−82				Gamel et al. (43)
PW	Bar	Baking	30 min at 149 °C	−47.3			+7.0	Gamel et al. (43)
PW	Crackers	Baking		−45.7			+12.6	Gamel et al. (43)
PDW	Pasta	Cooking andDrying	Series of steps	−74.5		−17.5	+6.3	Ficco et al. (13)
YDW	Pasta	Cooking andDrying	Series of steps			−19.2	+15.4	Ficco et al. (13)
PW	Biscuit	Baking	20 min at 160 C	−55.06	−38.4	−10.1	+6.8	Pasqualone et al. (11)
PW	Chapatti	Baking	1 min at 210 °C	−56	−47.5		−35.6	Kumari et al. (9)
BW	Chapatti	Baking	1 min at 210 °C	−31.3	−36.4		−16.1	Kumari et al. (9)
Bl–W	Chapatti	Baking	1 min at 210 °C	−29	−26.2		−14.9	Kumari et al. (9)
WF+PW Bran	Pasta	Baking		−58.2	−59.7			Parizad et al. (70)
WS+PW Bran	Pasta	Baking		−65.2	−51.1			Parizad et al. (70)
CW	Bread	steaming			−79.7		−21.5	Li et al. (71)
CW	Noodles	Baking			−70.1		−15.5	Li et al. (71)
PW	Bran	Baking	7–12 min at 177 °C	−10.4	+10.2	-	+13	Li et al. (12)
WB	Bran	Heating			+22.5			Calinoiu et al. (72)
PW	Infant cereals		30 min at 120 °C	−62.15	−24.14		+19.2	Hirawan et al. (73)
CW		Extrusion	130 °C			−74.3		Paznocht et al. (61)
CW		Puffing	270–280 °C for 6 Sec			−68.4		Paznocht et al. (61)

*TAC, total anthocyanin content; TYP, total yellow pigment; TPC, total phenolic acid content; YDW, yellow durum wheat; PDW, purple durum wheat; YDW, yellow durum wheat; TCC, Total carotenoid content; WB, bread wheat; CW, colored wheat; Bl-W, Black wheat; BW, Blue Wheat; PW, purple wheat*.

Abdel-Aal and Hulc (68) conducted initial studies on the effect of heat on anthocyanin-rich wheat flour and found degradation of anthocyanin content in blue wheat. Nevertheless, the degradation is less when compared to anthocyanin extracts; this might be due to the protective effect of the food matrix in the whole wheat flour. Several studies have reported reducing anthocyanin content upon cooking in colored wheat (11, 65). Bartl et al. (54) compared the effect of baking time and temperature on reducing anthocyanins during bread making from purple and blue wheat. Both purple and blue wheat lines showed a higher reduction in anthocyanin content with a longer baking time, even at a lower temperature. So, high temperature and short-duration baking are considered more anthocyanin-sparing than low temperature and long duration.

Further, the reduction rates were higher for purple wheat than blue wheat. Similarly, several other products were investigated. Chapatti making reduces anthocyanin content with a higher reduction in the anthocyanin content in a purple chapatti, followed by blue and then black (9). A comparison of different products, e.g., bread, pancakes, porridge, bars, and crackers from purple wheat, indicated that pancakes, bars, and crackers showed lower reduction than bread and porridge (43). Different bread-making stages on anthocyanin content in blue and purple wheat indicated that anthocyanins were lost during the baking process in blue wheat bread. However, in purple, the most significant losses were observed during dough making (69). Similarly, Ficco et al. (13) studied different pasta-making stages for purple durum wheat. Their results depicted that the drying process has a higher effect on the anthocyanin reduction of the pasta. Parizad et al. (70) compared the pasta prepared from purple wheat bran-enriched flour and semolina and found a higher reduction in anthocyanin content in the semolina-based pasta than the flour-based.

Other than anthocyanins, several reports have indicated changes in the TPC of colored wheat-based food products. Hirawan et al. (73) observed a reduction in TPC in the case of purple wheat infant cereals. The TPC content was lower in cooked pasta made from white wheat flour and semolina enriched with purple wheat bran by Parizad et al. (70). The TPC content of purple wheat cookies was likewise reduced according to Pasqualone et al. (11). The TPC and TFC levels were similarly shown to be lower after preparing noodles and steamed bread according to (71). In contrast, Calinoiu et al. (72) have observed an increase in the TPC of thermally processed wheat bran compared to fresh wheat bran samples. Yu and Beta (65) showed that the TPC increased mixing, fermenting, and baking processes. The increase in TPC during baking processes is due to Maillard reaction products because mixing, proofing, and baking had a minor effect on the total phenolic content. Because of Maillard reaction products, baking slightly increased the concentration of phenolics in a bread crust. The bread crust contained the highest TPC, followed by whole bread and bread crumbs (74).

Carotenoids changes in response to product-making were also observed in colored wheat. Ficco et al. (13) reported that the extrusion and drying process reduced the TYC in purple and yellow durum wheat pasta. Paznocht et al. (59) reported a reduction in the total carotenoid content (TCC) of extruded and puffed products made of colored grain wheat. Moreover, they discovered that puffing conserves more carotenoid than extrusion.

Besides measuring the changes in TAC, TPC, and TYC, antioxidant activity after product-making is vital for assessing the functionality of food products for their commercial use. Pasqualone et al. (11) reported an increase in the antioxidant activity of purple wheat biscuits. Similarly, Hirawan et al. (73) also observed an increase in the antioxidant activity of infant purple wheat cereals. Ficco et al. (13) reported an increase in the antioxidant activity of yellow and purple durum wheat pasta. Gamel et al. (43) reported an increase in the antioxidant activity of purple wheat flakes, bars, and crackers compared to its flour. Li et al. (12) observed increased antioxidant properties of thermally processed purple wheat bran and muffin. Yu and Beta (65) studied the effect of baking on the antioxidant activity of the bread prepared from purple wheat and observed an increase in the antioxidant activity. In contrast, Kumari et al. (9) reported a decrease in the antioxidant activity in purple, blue, and black chapattis and Li et al. (71) in the case of bread and noodles. Both studies reported a lower reduction in the antioxidant activity than the reduction in the anthocyanin or phenolic contents.

Most of the studies indicated that although there is a decrease in the anthocyanin content during the product-making processes (mixing, kneading, fermentation, and baking) in colored wheat, still, there is either an increase or a relatively lower decrease in the antioxidant activity of the final product. The possible reason might be that the breakdown products of anthocyanins and phenolic compounds after heating might have higher antioxidant activity than their colored native forms, or this might be a synergistic effect of different phytochemicals. However, more research is necessary to determine the effects of food processing on food functionality to develop processing approaches that would better retain these bioactive compounds in colored wheat final products.

## Effect of Genotype and Environment on Anthocyanin Accumulation in Colored Wheat

The anthocyanin content is affected by environmental factors like UV radiation, temperature, wounding, infection by pathogenic fungi, phytohormone, sugar, ions, magnesium fertilization, the position of grain in spike, sowing date, and nitrogen application (21, 53, 58, 75, 76). The accumulation of anthocyanins in colored wheat grain starts toward the middle of grain filling. Therefore, the temperature at this stage influences anthocyanin accumulation. The lower temperature at the time of harvest favors anthocyanin accumulation. Purple and blue wheat for Saskatchewan, Canada, with 19/5°C day-night temperature, has higher anthocyanin content than 35/18°C for Mohali, India (**Table 4**). Similarly, black wheat cultivated at 24/17 °C in Tainan, China, has higher anthocyanin content than 35/18°C in India (Table 3). Although, the anthocyanin content for blue wheat is not much different in the case of 22/15°C at Taian, China, 20/9°C at Kromeriz, Czech Republic, or 35/18°C for Mohali, India (Table 4). Thus, genotype also has a significant influence on the anthocyanin content. Three genotypes of purple wheat from Denmark and two from Canada showed considerable differences in the anthocyanin content (Table 4). Thus, the final anthocyanin content in the seed depends on the genotype and environment interaction.

**Table 3:** Effect of genotype and environment on the anthocyanin accumulation in colored wheat.

**Wheat color**	**Wheat variety**	**TAC (mg/kg)**	**Environment**	**Sowing temperature**	**Harvesting temperature**	**References**
Blue	NABIMG-9	80.3	Mohali, India	26/12 °C	35/18 °C	Kumari et al. (9)
	L58	72.4	Taian, China	23/16 °C	22/15 °C	Wang et al. (57)
	Sebesta Blue	118.4	Foggia, Italy	24/16 °C	22/13 °C	Ficco et al. (17)
						
	Skorpion	72	Kromeriz, Czech Republic	13/4 °C	20/9°C	Giordano et al. (66)
	Purendo	211.9	Saskatchewan, Canada	23/9 °C	19/5 °C	Abdel-Aal et al. (18)
						
	Purendo38	75.7	Saskatchewan, Canada	23/9 °C	19/5 °C	Knievel et al. (21)
	PIG99006	105.7		23/9 °C	19/5 °C	
						
Purple	NABIMG-10	43.8	Mohali, India	26/12 °C	35/18 °C	Kumari et al. (9)
	Rosso	102	Austria	21/12°C	26/16 °C	Giordano et al. (66)
	Indigo	91.7	Belgrade, Serbia	18/8°C	25/19 °C	Žilić et al. (55)
	Durum Desf	22.6	Foggia, Italy	24/16 °C	22/13 °C	Ficco et al. (17)
	Charcoal	234.5	Jutland, Denmark	16/9 °C	20/13 °C	Liu et al. (56)
	Konini	25.4				
	Indigo	72.4				
	Laval	95.8	Saskatchewan, Canada	23/9 °C	19/5 °C	Abdel-Aal et al. (18)
	Konini	38		23/9 °C	19/5 °C	
	PIG03008	278	Saskatchewan, Canada	23/9 °C	19/5 °C	Knievel et al. (21)
	Laval-19	51.8		23/9 °C	19/5 °C	
Red	Durum Desf	9.9	Foggia, Italy	24/16 °C	22/13 °C	Ficco et al. (17)
	Red Fife	9.6	Copenhagen, Denmark	16/9 °C	20/13 °C	Liu et al. (56)
	Katepwa	7.9	Saskatchewan, Canada	23/9 °C	19/5 °C	Abdel-Aal et al. (18)
	Freedom	6.7	Guelph, Canada	23/9 °C	19/5 °C	
Black	NABIMG-11	140.1	Mohali, India	26/12 °C	35/18 °C	Kumari et al. (9)
						
	YD	277.6	Taian, China	17/1 °C	24/17 °C	Wang et al. (57)

**Table 4:** Compilation of *in vitro* and *in vivo* studies indicating the positive effect of colored wheat on human health.

**S. No**.	**Country**	**Wheat**	**Model**	**Method**	**Extraction Solvent**	**Effect**	**References**
1	Russia	Purple (grain)	Mouse model of Alzheimer's disease	NA	NA	Prolonged memory extinction; As functional nutrition at the early stages of neurodegenerative disorders	Tikhonova et al. (77)
2.	India	Purple, Black	Mouse model of high fat diet	NA	NA	Reduction in body weight gain, fat pad, total cholesterol, triglyceride, free fatty acid levels in serum, restoration of blood glucose insulin resistance, activation fatty acid-β oxidation glucose insulin resistance, activation fatty acid-β oxidation	Sharma et al. (52)
			Mouse model oxidative stress	MDA, SOD, GSH, IL6,TNFα, CRP	NA	High anti-oxidant activity in liver	
3	Canada	Purple (Bars)	Obese adults with chronic inflammation	GSH, ox-LDL, IL6,TNFα, CRP	1.5% HCl in ethanol	Anti-inflammatory anti-oxidant capacity	Gamel et al. (78)
4	India	Purple, Blue, Black (Chapatti)	NA	DPPH, ABTS, PCL	1.5% HCl in methanol	High dietary fibers, protein content, phenolic compounds, anthocyanin content, antioxidant activity	Kumari et al. (9)
5	Italy	Blue (Sourdoughs)	Murine macrophage cell line RAW 264.7	FRAP, ABTS	1.5% HCl in ethanol	High anti-oxidant activity in cultured cells	Galli et al. (79)
6	India	Purple, Blue, Black (Flour, wheat grass)	NA	DPPH, ABTS	1.5% HCl in methanol	Antimicrobial activity of black wheat against bacteria yeast, Antioxidant activity	Sharma et al. (80)
7	Italy	Purple (Pasta)	Human intestinal epithelial Caco-2 cells	Enzyme activity; NF-κB	1% HCl in Ethanol	α- glucosidase, α-amylase inhibition, anti-inflammatory activity	Parizad et al. (70)
8	Canada	Purple (Bars, Crackers)	NA	ABTS, DPPH, ORAC	1.5% HCl in ethanol	High anti-oxidant activity, protein content, Dietary fiber	Gamel et al. (78)
9	China	Purple	NA	NA	NA	High Se	Xia et al. (46)
10	South Korea	Purple (Flour)	NA	DPPH, APX, Catalase, SOD, Peroxidase	1% HCl in methanol, Phosphate buffer	Anti-oxidant activity	Hong et al. (81)
11	China	Purple, Blue, Black (Flour)	NA	DPPH, ABTS	1% HCl in Ethanol	High Antioxidant activity	Wang et al. (57)
12	Canada	Purple	Normal Human	NA	NA	Positive effects on digestion, metabolism, utilization of the products	Gamel et al. (43)
13	Czech Republic	Purple	NA	NA	NA	High yellow pigment content	Paznocht et al. (59)
14	India	Purple, Blue, Black (Flour)	Murine macrophage cell line RAW 264.7	DPPH, ABTS, PCL	1.5% HCl in ethanol	High anti-oxidant anti-inflammatory activity, High Fe, Zn	Sharma et al. (34)
15	Czech Republic	Blue, Purple (Flour)	NA	DPPH	0.1% HCl in methanol	High anti-oxidant activity	Zrckova et al. (82)
16	Czech Republic	Blue (Pellets)	Rat	FRAP	1% HCl in methanol	Increases anti-oxidant status of rat blood in vivo influenced the drug- metabolizig microsomal cytochromes P450	Prokop et al. (83)
17	China	Purple (Flour)	NA	FRAP	Methanol/acetone/ water solution (7:7:6)	High protein content, phenolics,Fe, Zn anti-oxidant activity	Ma et al. (38)
18	China	Black	Patients with type 2 diabetes	NA	NA	Improves glycemia inflammatory profile of type 2 diabetes mellitus (T2DM) patients	Liu et al. (84)
19	Slovakia, Ukraine, Poland, Germany	Purple (Flour, wheat grass)	NA	DPPH, ABTS	1% HCl in Methanol	High anti-oxidant activity	Sytar et al. (85)
20	Canada	Purple (Bran Flour)	NA	DPPH, TEAC, ABTS	1.5% HCl in ethanol	High anti-oxidant activity	Abdel-Aal et al. (35)
21	China	Blue, Blue, Black	NA	NA	NA	High protein, essential amino acids content, lysine, Zn, Fe, Mg	Tian et al. (39)
22	Ethiopia	Purple (Flour)	NA	NA	1.5% HCl in methanol	High anthocynin content	Kassegn (37)
23	Russia	Purple (Biscuits)	NA	Blizar antioxidant activity analyzer	1% aqueous solution of HCl	High anti-oxidant activity	Khlestkina et al. (67)
24	Czech Republic	Purple, Blue	NA	NA	NA	High yellow pigment content	Paznocht et al. (60)
25	Czech Republic	Purple	NA	NA	NA	Vitamin E activity	Lachman et al. (49)
26	Czech Republic	Purple, Blue	NA	NA	NA	Vitamin B1, B2, B6, E	Granda et al. (50)
27	China	Blue	NA	NA	NA	High phenolics, High antioxidant activity	Zhang et al. (86)
28	Russia	Bread	NA	Amperometric method		High anti-oxidant activity	Khlestkina et al. (67)
29	Czech Republic	Purple	Rats, chickens, fish	DPPH, FR, FRAP, ABTS	NA	Improves anti-oxidant activity function of the liver tissue	Mrkvicová et al. (87)
30	Italy	Purple, Blue (Milling fractions)	NA	DPPH	1.5% HCl in ethanol	Anti-oxidant activity	Giordano et al. (66)
31	Slovakia	Purple	Rats	AOPP, TBARS, AGEs, FRAP	NA	Anti-oxidant activity (Serum)	Janšáková et al. (88)
32	Italy	Purple (Pasta)	NA	NA	NA	Low glycemic index, High yellow pigment content	Ficco et al. (13)
33	Italy	Purple (biscuits)	NA	DPPH	1.5% HCl in methanol	High protein, anti-oxidant activity	Pasqualone et al. (11)
34	Canada	Purple (bread)	NA	DPPH, ABTS	1.5% HCl in methanol	High anti-oxidant activity	Yu and Beta (65)
35	China	Purple, Black (Noodles, Bread)	NA	FRAP. ABTS	1.5% HCl in methanol	High phenolics, high anti-oxidant activity	Li et al. (71)
36	Slovakia	Purple (Milling fractions)	NA	DPPH	Methanol	High anti-oxidant activity	Ivanišová et al. (89)
37	Italy	Blue, Purple (Flour)	NA	TEAC	1.5% HCl in methanol	High Fe, Zn Mn, Anioxidant activity	Ficco et al. (17)
38	China	Black	NA	ABTS	NA	High total phenolic, carotenoid favonoidcontents, anti-oxidant activity	Sun et al. (63)
39	China	Purple (Flour)	NA	NA	NA	High protein content, Zn, Fe, Mg, K, Vitamins B2, B9, E	Guo et al. (40)
40	Germany	Purple	*Caenorhabditis elegans*	DPPH	1.5% HCl in methanol	Extended mean life span, high anti-oxidant activity	Chen et al. (90)
41	United States	Blue	NA	FC, TEAC, LMB	80% ethanol, acetone, ethyl acetate	Anti-oxidant activity	Tyl and Bunzel (91)
42	Canada	Purple (Infant cereals)	Primary human fetal small intestine cell line (FHs 74 Int, CCL-241)	ORAC	1.5% HCl in methanol	High anti-oxidant activity	Hirawan et al. (73)
43	Ethiopia, Vienna, Austria	Blue, Purple	NA	NA	1.5% HCl in methanol	High Anthocyanin protein content	Eticha et al. (92)
44	Canada, China	Purple	NA	DPPH, ORAC	1.5% HCl in methanol	Anti-oxidant activity, Total flavonoid content	Liu et al. (56)
45	Canada	Blue	NA	DPPH, ABTS, LDL Oxidation	1.5% HCl in ethanol	Anti-oxidant activity	Abdel-Aal et al. (58)
46	Canada, China	Black	NA	NA	NA	High protein	Nandy et al. (93)
47	Canada	Purple	NA	NA	NA	High Melatonin	Hosseinian et al. (19)
48	Canada, Hong Kong, China	Blue	Mouse macrophage RAW264.7 cell	DPPH, ABTS, ORAC	0.1% HCl in methanol	Anti-oxidant activity	Hu et al. (94)
49	Austria, Italy	Purple, Blue	NA	TEAC, FRAP	Citric Acid + Ethyl Acetate + Methanol	Anti-oxidant activity	Siebenhandl et al. (95)
50	China, Canada	Purple (Muffins)	NA	ORAC, DPPH	1.5% HCl in ethanol	Anti-oxidant activity	Li et al. (10)
51	Canada	Purple	NA	ORAC, DPPH	1.5% HCl in ethanol	High anti-oxidant activity	Li et al. (12)
52	Canada, China, Hong Kong	Black	NA			High Protein content, total essential amino acids, total amino acids, content, High Se	Li et al. (41)
53	China, Canada, Hong Kong	Black	NA	DPPH	Methanol (100%)	High Anti-oxidant activity	Li et al. (29)
54	China	Purple	NA	NA	NA	High Fe, Zn	He and Ning (45)

*ABTS, 2,2′-azino-bis(3-ethylbenzothiazoline-6-sulfonic acid); DPPH, 1,1 diphenyl-2-picrylhydrazyl; FC, Folin–Ciocalteu; FRAP, Ferric reducing ability of plasma; FR, Free Radicals method; LMB, Leucomethylene blue; ORAC, Oxygen radicals absorbance capacity; PCL, Photochemiluminescence; TEAC, Trolox equivalent antioxidant capacity method; MDA, Malondialdehyde test; SOD, Superoxide dismutase; GSH, Glutathione peroxidase; ox-LDL, Oxidized low-density lipoprotein; TNFα, Tumor necrosis factor α; CRP, C-Reactive Protein; AOPP, Advanced oxidation protein products; TBARS, Thiobarbituric acid reactive substances; AGEs, Advanced glycation end products; NF-κB, Nuclear factor kappa-light-chain-enhancer of activated B cells; APX, Ascorbate peroxidase*.

## Applications of Colored Wheat in Health

Anthocyanins are considered biologically active compounds and play a vital role in preventing several metabolic diseases; thus, they have been hailed as a nutraceutical agent. They are potent antioxidants due to exceptionally high radical scavenging activities (6). Thus, anthocyanins perform a panorama of biomedical functions (5, 7, 8). Numerous epidemiological studies have already established the anti-proliferative, antioxidant, antiaging, and anti-inflammatory properties of anthocyanins from diverse sources (5, 6). Anthocyanins are characterized by various colored cereals like rice, sorghum, barley, maize, and quinoa (50, 96). Recently much attention was focused on colored wheat varieties. Several studies have reported the health benefits of colored wheat, associated with its high anthocyanin content, phenolic content, and antioxidant activity.

Research publications from over 60 institutes from 16 countries mentioned the positive effects of colored wheat on health (Table 3). Since 1999, several studies from India, South Korea, China, Canada, Ethiopia, Italy, and Austria have reported on the health benefits of colored wheat flour and bran. Since 2015, numerous organizations from India, Canada, Slovakia, Ukraine, Poland, Germany, Russia, Italy, and China have reported on the functioning of various bakery items such as bread, biscuits, and chapatti. Different institutions in Russia, India, the Czech Republic, Germany, Canada, Hong Kong, China, and Italy are experimenting on cell lines and animal models. They all support colored wheat's health advantages. Furthermore, two separate human studies were done in China and Canada in 2018 and 2020, indicating that anthocyanin-rich wheat types are used as a functional food.

### *In vitro* Studies Supporting the Antioxidant Activity of Colored Wheat

Around 30 studies have reported higher antioxidant activity of colored wheat than white wheat (Table 3). Authors have used single or multiple *in vitro* assays to assess the antioxidant activity. Seven studies have used DPPH (1,1 diphenyl 2 picrylhydrazyl) assay for estimation of antioxidant activity and reported higher antioxidant activity of colored wheat extracts in comparison to white wheat (11, 29, 66, 82, 89, 90). Apart from DPPH, several other assays have been used singly for the assessment of antioxidant activity of colored wheat, such as TEAC (Trolox equivalent antioxidant capacity), ABTS (2,2′ azinobis (3 ethylbenzothiazoline 6 sulfonic acid), ORAC (oxygen radicals absorbance capacity), FRAP (ferric antioxidant power) (17, 38, 63, 83). Six studies have used two assays that include DPPH + ABTS / FRAP + ABTS / DPPH + ORAC/ TEAC + FRAP, and all have reported high antioxidant activity of colored wheat lines in comparison to white wheat (10, 52, 56, 57, 65, 79, 85, 95). Several studies have used three or more than three assays, e.g., DPPH, ABTS, and PCL (photochemiluminescence) / ABTS, DPPH, ORAC / DPPH, TEAC, ABTS / FC (Folin–Ciocalteu), TEAC, and LMB (leucomethylene blue assays) to report higher antioxidant activity of colored wheat compared to white wheat (9, 35, 88, 91). Hong et al. (81) used enzyme activities of selected antioxidant-related proteins, including APX (ascorbate peroxidase), catalase, SOD (superoxide dismutase), and peroxidase to report higher antioxidant activity of purple wheat than white wheat. Abdel-Aal et al. (58) used copper-induced human LDL (low-density lipoprotein) oxidation and found higher antioxidant activity of blue wheat bran. Among different colored lines, the highest antioxidant activity has been observed in black wheat and follows the trend of black> blue> purple> white (9, 34, 52). High antioxidant activity in the colored wheat grain is seen in bran than in flour due to anthocyanins in the seed coat (35, 66, 89). Wheatgrass (sprouts) from different colored wheat lines also synthesizes anthocyanins. Studies carried out on colored wheatgrass indicated that black wheatgrass has the highest antioxidant activity, followed by purple and then blue, and least in the case of white wheatgrass (black> purple> blue> white) (52, 85). Apart from colored wheat grain, flour or bran, antioxidant activity has also been assessed for different food products. Higher antioxidant activity of colored wheat products than white has been observed for biscuits (11), chapatti (9), bars and crackers (36), bread (65, 67), noodles, steamed bread (71), muffins (12), and infant cereals (73) (Table 3). The antioxidant activity in colored wheat is mainly associated with its higher anthocyanin content. However, a study conducted by Morgounov et al. (97) revealed that the antioxidant activity of purple wheat was not consistently higher than white wheat across environments. The reason is a method used for the estimation. Authors have measured the antioxidant activity (ABTS) directly from the flour without extraction with acidified methanol as done in the case of more than 30 studies mentioned above (Table 3). Moreover, there might be chances of interference of several components in the flour like phenolics, tocopherols, and carotenoids, contributing to the total antioxidant activity. Moreover, acidification (low pH) of solvent is essential for the efficient extraction of anthocyanins. Tyl and Bunzel (91) found that anthocyanins were significant contributors to blue wheat antioxidant activity only upon extraction under acidic conditions.

### Cell Lines-Based Studies Supporting the Positive Effect of Colored Wheat on Human Health

In an *in vitro* cell line-based study by Hu et al. (94), the antioxidant activity of blue wheat was assessed for its affinity to reducing both reactive oxygen species (ROS) and reactive nitrogen species (RNS). The intracellular oxidation initiated by hydrogen peroxide in mouse RAW264.7 macrophage cells was suppressed upon the addition of blue wheat bran extract. Also, nitric oxide production in endotoxin (lipopolysaccharides-{LPS})-activated macrophage cells was significantly suppressed by the blue wheat extract, thus indicating suppression of both ROS and RNS activity and contribution to an anti-inflammatory effect in addition to an antioxidant property (Table 3). The report published by Sharma et al. (34) used the RAW 264.7 cell lines to study the effect of three types of anthocyanin-biofortified wheat (purple, blue, and black). The production of nitric oxide and pro-inflammatory cytokines produced by LPS induction was attenuated upon treatment of cell lines with the anthocyanin extracts from the colored wheat. The purple wheat extract showed the highest anti-inflammatory effect and followed the trend of white<blue<black<purple. *In vitro* study conducted by Hirawan et al. (73) investigated the antioxidant potential of purple wheat-based infant cereals using primary human fetal small intestine cell line FHs 74Int. Purple wheat cereals showed higher cellular antioxidant activity than commercial infant cereals. Galli et al. (79) used RAW 264.7 murine macrophage cell lines to study the antioxidant property of sourdoughs made from blue wheat. Blue wheat sourdoughs had more significant antioxidant activity than that recovered in white wheat despite inoculation with the same lactobacilli strains. Overall, all cell line-based studies indicated the antioxidant and anti-inflammatory effects of colored wheat.

### Animal and Human Studies Supporting the Positive Effect of Colored Wheat on Human Health

Dietary intake of anthocyanin-rich extract from different fruits and vegetables has proven its beneficial effects through several animal and human studies (5–8). Certain reports have also demonstrated the effect of anthocyanin-rich cereal grains through various *in vivo* studies in combating various disorders (77). From the cereal grains, colored wheat has emerged in many scientific publications, and various *in vivo* and human studies are also available supporting their role in health. Anthocyanin-rich black wheat (NABIMG-11) significantly reduced the body weight gain and fat pad in the *in vivo* study carried out on a high-fat diet-induced (HFD) mouse model (80). Further, black wheat and purple wheat (NABIMG-10) reduced total free fatty acid, triglyceride, and cholesterol levels in serum, along with the restoration of insulin resistance and blood glucose. Furthermore, colored wheat was tested for its anti-oxidative properties in an experimental mouse stress model. It indicated that black wheat acted as an *in vivo* antioxidant, effectively lowering oxidative stress markers like MDA (malondialdehyde test), SOD, and GSH (glutathione peroxidase) (80). Purple wheat (i:S29Pp-A1Pp-D1Pp3P) study on a mouse model of Alzheimer's disease and a transgenic mouse model of Parkinson's disease (PD) indicated that an anthocyanin-rich diet was safe and possessed positive effects on cognitive function (77). Anthocyanins in purple wheat prevented deficits in working memory induced by Alzheimer's disease. The results suggested that anthocyanin-rich wheat is a promising source of functional nutrition in the early stages of neurodegenerative disorders. A study of blue wheat varieties (UC66049 and Skorpian) on normal rats showed a positive effect on the antioxidant status of plasma and cytochrome P450 levels in the liver (83). Similarly, purple wheat (Karkulka) was assessed to improve rats' oxidative status and behavior. Anthocyanin-rich purple wheat positively affected serum antioxidant status and kidney protein oxidation. However, effects like increased lipid peroxidation in the kidney and modified animal behavior related to anxiety were also observed (88). Purple (Konini) wheat proved beneficial in improving the liver antioxidant activity and liver function enzyme activities in rats and chickens (87). However, the effect was not observed in a similar study carried out on fishes; the reason could be that fishes could not metabolize the purple wheat properly (87). Besides this, purple wheat extends the life span and reduces the oxidative stress of wild-type and mutant forms of *Caenorhabditis elegans* (90). Further, 15% of purple wheat did not affect the oxidative stress in the rabbits (98). This 15% quantity of purple wheat may be too little to affect the oxidative parameters.

Human studies have also shown that black grain wheat positively affected type 2 diabetic subjects by alleviating the glycated albumin levels and inflammatory markers like TNF-α and IL-6 with no difference in blood glucose and insulin levels (84). A study on healthy individuals indicated that bran-enriched purple wheat bars or crackers had positive effects on digestion, metabolism, and utilization of the products even though very small or no changes in plasma antioxidant activity or inflammatory biomarkers were observed (43). In another study on overweight and obese adults, purple wheat bars were modestly efficacious in improving the plasma antioxidant status, reducing the fasting glucose concentration, adiponectin levels, and inflammation (78).

Overall, as expected, both animal and human investigations using experimental models or non-communicable disease conditions showed a significant effect of colored wheat in ameliorating the antioxidant status and associated health conditions, while the effects observed in normal animals and humans were comparatively less pronounced. These observations indicate the positive effects of colored wheat in diminishing the risk of lifestyle-associated disorders and chronic ailments. Further, black wheat exhibited higher effects than blue and purple wheat, which may be associated with its higher anthocyanin content.

### Other Studies Supporting the Positive Effect of Colored Wheat on Human Health

Sharma et al. (52) identified the antimicrobial property of colored wheat extracts against harmful human pathogens. They compared different colored wheat lines and found that black wheat showed higher antimicrobial activity, followed by purple, blue, and white wheat. Sharma et al. (42) reported that anthocyanins in colored wheat have cooking-associated amino acid losses prevention capability.

### Breeding and Agronomic Traits of Colored Wheat

The major challenge for a new variety of colored wheat is grain yield at harvest. Breeding efforts have indicated that grain yield is the major hurdle in the popularization of colored wheat lines because of the linkage drag associated with the blue aleurone trait contributed by the wild wheat in the form of addition, substitution, or translocation lines (4, 25). Rigorous breeding is required to disrupt these linkages to create lines with high anthocyanin content and a satisfactory yield level. Skorpion, a commercial blue grain cultivar from Austria, had a 25% lower yield than check cultivars (25). The lower yield of four different colored wheat cultivars was reported under the organic cropping system (82). The yield of purple wheat isogenic lines of S29, although comparable to the S29, was found to be less than half of the commercial cultivar Seri (97). Breeders are making selections from the available colored wheat germplasm for use in the breeding program (31). Breeding efforts have increased the yield and agronomic parameters of different breeding lines/cultivars compared to donor-colored wheat lines (4, 99–102).

The other important trait is adaptability to the environment. Morgounov et al. (97) reported a more significant environmental effect than the genotype for purple wheat productivity. Further, native blue-colored wheat varieties are winter wheat in nature. It needs a prolonged vegetative phase for biomass production, but when transferred to the subtropical region, they experience multiple stresses because of poor adaptability. Garg et al. (4) demonstrated that color wheat varieties adapted to various environments by using breeding strategies like crossing exotic winter color wheat lines to locally adapted spring wheat cultivars. They have generated colored wheat lines for the Indian environment having high anthocyanin content and a better yield (4). In another study, two different isogenic lines for purple grain (Purple, Purple Feed) in the background of cv. S-29 were studied. The lines originating from Purple Feed had substantially improved the grain yield and productivity compared to S-29 (97). Different colored wheat cultivars were also investigated under the effects of combined nitrogen (N) and phosphorous (P) fertilization. High N application increased grain yield (38). Fan et al. (75) have identified various colored wheat genotypes which showed tolerance to low-nitrogen conditions. Besides, it was demonstrated that under salt and phosphate stress, colored wheat genotypes could maintain significantly higher dry matter production (48, 103). Therefore, the development of high-yielding-colored varieties resistant to diseases and biotic and abiotic stresses would play a significant role in transfiguring the perception by the public that can result in their adoption across the globe.

## Challenges Ahead

Although colored wheat is a research trend, its commercialization is still a challenge. The area under cultivation of colored wheat is less than 0.01% of the total area under wheat. In an online search, only a few colored wheat products were found, including PurPur purple wheat bread from Austria, Koka purple wheat noodles from Singapore, Dayspring, Antho grains, and several other brands, whole black and purple wheat flour, and porridge (Dalia) from India, and Gardenia purple wheat bread from Canada. The major challenge for the wide-scale adoption is market generation and consumer awareness creation. For this, government programs and industry should come forward and join their hands for this healthier option of wheat.

In addition, there is enormous research potential, e.g., innovative product development, large-scale human trials for functional validation, breeding for improvement of anthocyanins, additional phytonutrients like lutein, tocopherols, proteins, minerals including Fe, Zn, Se, and biotic and abiotic stress tolerance and yield.

## Conclusion

Colored wheat, particularly black wheat, has been a popular study topic that can be turned into high-quality commercial foods. It is an excellent source of bioactive phytochemicals with preventive effects against inflammation, metabolic syndrome, obesity, diabetes, dyslipidemia, aging, and neurodegeneration. Cooking the colored wheat reduces anthocyanin and other phytochemicals content but does not reduce the antioxidant activity. It is expected to become a trendy functional food in many countries eventually. However, the generation of market and consumer awareness is the crucial challenge of its large-scale commercialization. There is a need to develop end-product-oriented cultivars based on regional preferences with high yield, anthocyanin content, carotenoids, phenolics, vitamins, and minerals and associate functionality with them by *in vitro* and *in vivo* studies.

## Author Contributions

MG: conceptualization, validation, supervision, project administration, and writing–review and editing. SK: writing–original draft, review, and editing. AS, AK, VT, SS, PK, and BS: writing–data curation, original draft preparation, and editing. All authors contributed to the article and approved the submitted version.

## Funding

This work was supported by the National Agri-Food Biotechnology Institute, Mohali, Punjab, India.

## Conflict of Interest

The authors declare that the research was conducted in the absence of any commercial or financial relationships that could be construed as a potential conflict of interest.

## Publisher's Note

All claims expressed in this article are solely those of the authors and do not necessarily represent those of their affiliated organizations, or those of the publisher, the editors and the reviewers. Any product that may be evaluated in this article, or claim that may be made by its manufacturer, is not guaranteed or endorsed by the publisher.
